# How do red and infrared low-level lasers affect folliculogenesis cycle in rat’s ovary tissue in comparison with clomiphene under in vivo condition

**DOI:** 10.1007/s10103-017-2296-5

**Published:** 2017-08-11

**Authors:** Paria Naseri, Alireza Alihemmati, Seyed Hossein Rasta

**Affiliations:** 1 Department of Medical Physics and Department of Medical Bioengineering, Tabriz University of Medical Sciences, Tabriz, Iran; 2 Stem Cell Research Centre, Tabriz University of Medical Sciences, Tabriz, Iran; 3 Department of Anatomical Sciences, Histology and Embryology, Faculty of Medicine, Tabriz University of Medical Sciences, Tabriz, 51666 Iran; 4 School of Medical Sciences, University of Aberdeen, Aberdeen, UK

**Keywords:** Near-infrared laser, Red laser, Clomiphene drug, Folliculogenesis cycle, Polycystic ovarian syndrome, Rat ovary tissue

## Abstract

Folliculogenesis is a cycle that produces the majority of oocyte. Any disruption to this cycle leads to ovulation diseases, like polycystic ovarian syndrome (PCOS). Treatments include drugs and surgery; lasers have also been used complementarily. Meanwhile, still there is no definite treatment for PCOS. This study investigated the photo-bio stimulation effect of near-infrared and red low-level laser on producing follicles and compared the result with result of using common drug, clomiphene. Therefore, the aim of this study was to propose the use of lasers autonomously treatment. So, there was one question: how do lasers affect folliculogenesis cycle in rat’s ovary tissue? In this study, 28 rats were assigned to four groups as follows: control (CT), clomiphene drug (D), red laser (RL), and near-infrared laser (NIRL). Afterwards, 14 rats of RL and NIRL groups received laser on the first 2 days of estrous cycle, each 6 days, for 48 days. During treatment period, each rat received energy density of 5 J/cm^2^. Seven rats in D group received clomiphene. After the experiment, lasers’ effects at two wavelengths of 630 and 810 nm groups have been investigated and compared with clomiphene and CT groups. Producing different follicles to complement folliculogenesis cycle increased in NIRL and RL groups, but this increase was significant only in the NIRL group. This indicates that NIRL increases ovarian activity to produce oocyte that certainly can be used in future studies for finding a cure to ovarian negligence to produce more oocyte and treat diseases caused by it like PCOS.

## Introduction

Folliculogenesis is a cycle in which primordial follicles grow and produce graffian follicles in several stages and eventually produce the majority of oocyte [1]. Primordial follicles are embryonic [2] since there were a million of them resting follicle in every woman’s body at birth [3]. So, before puberty, mammalian ovary contains these follicles [4]. There are a lot of non-growing primordial follicles in their ovary (oocytes surrounded by flattened pre-granulosa cells) [5] most of which remain until puberty; then, they may either degenerate or activate and grow towards primary, secondary, tertiary, and quaternary stages (with an antral cavity) [6]. Following activation, primordial follicles develop and give rise to primary follicles (i) and then secondary follicles (ii). Next, by the stimulation of gonadotropin (FSH and luteinizing hormone (LH)), multi-layered antral follicles containing antral cavities are produced (iii); in the last stage, granulosa cells are demarcated and cause a single large antral cavity to be formed and the oocyte, ready for ovulation, reaches its final growth in the graffian follicle (iv); in other words, folliculogenesis cycle is completed [1, 7, 8]. Disorder in folliculogenesis cycle causes several diseases; polycystic ovarian syndrome (PCOS) is an important one [9]. In the reproductive age, 5 to 10% of reproductive age woman with PCOS are infertile [10, 11]. Search for a definite treatment of PCOS started in the early 1930s and still continues [11, 12]. The available modalities for induction of ovulation are weight reduction, clomiphene citrate, gonadotropin therapy, insulin sensitizer as metformin, and finally, ovarian drilling using laser or bipolar diathermy. Despite these treatment modalities, there are still cases resistant to treatment; this raises the need for a new treatment method [9, 13–15].

Different lasers can be used for PCOS drilling including argon, KTP, ND:YAG, and CO_2_. All of these stimulate ovary to increase activation of folliculogenesis cycle to ovulation. Lasers are also used to treat ovarian malignant and cancer [16, 17–19]. Degrees of response are variable [9].

Lasers have already been used as complement therapies, applied with medication or surgery, but they are also effective for ovarian stimulation [11, 16, 17, 20]. The direct effect of lasers, alone, on folliculogenesis cycle has not been investigated. So, the purpose of this study is to investigate the effect of lasers without surgery or drug on folliculogenesis cycle.

## Method and materials

### Animals

Initially, 28 female Wistar rats which have reached sexual maturity, with the average age of 9 weeks and in weight range of 150–300 g, were obtained from animal’s house of Tabriz University of Medical sciences (TUMS), Medical Physics Department. Then, rats’ general health was examined by estrous test in order to be in the same phase of menstrual cycle. They were randomly assigned to four groups (*n* = 7): control group (CT), without any intervention; clomiphene drug group (D), stimulated by clomiphene; red laser group (RL), stimulated by visible laser in red spectrum; and near-infrared laser (NIRL), stimulated by laser in the near-infrared spectrum. Afterwards, they were taken care of for 1 week on environmental conditions with free access to food and water; temperature (25–27 °C) and humidity were kept steady so that the rats would adapt to conditions of the new place. After a week, rats were weighed to ensure they remain in the range. Their behavior in the cage was examined during the experiment. The research protocol of this study was approved by Ethics in Research Committee of Tabriz University of Medical Sciences TUMS, under code number: TBZMED.REC.1394.238.

### Preparation process

Rats were painted for each group and labeled from one to seven. Then, their hairs in RL and NIRL groups were shaved and then the shaved areas were washed using Betadine. The point for laser radiation on their skin was determined and marked by marker. During the experiment, rats were in their own cages, expecting intervention or box cleaning (three times per week). Intervention on rats was done at the beginning of menstrual cycle. The preparation method in this study was designed based on references [9, 11, 20–25].

### Laser treatment

**Fig. 1:**
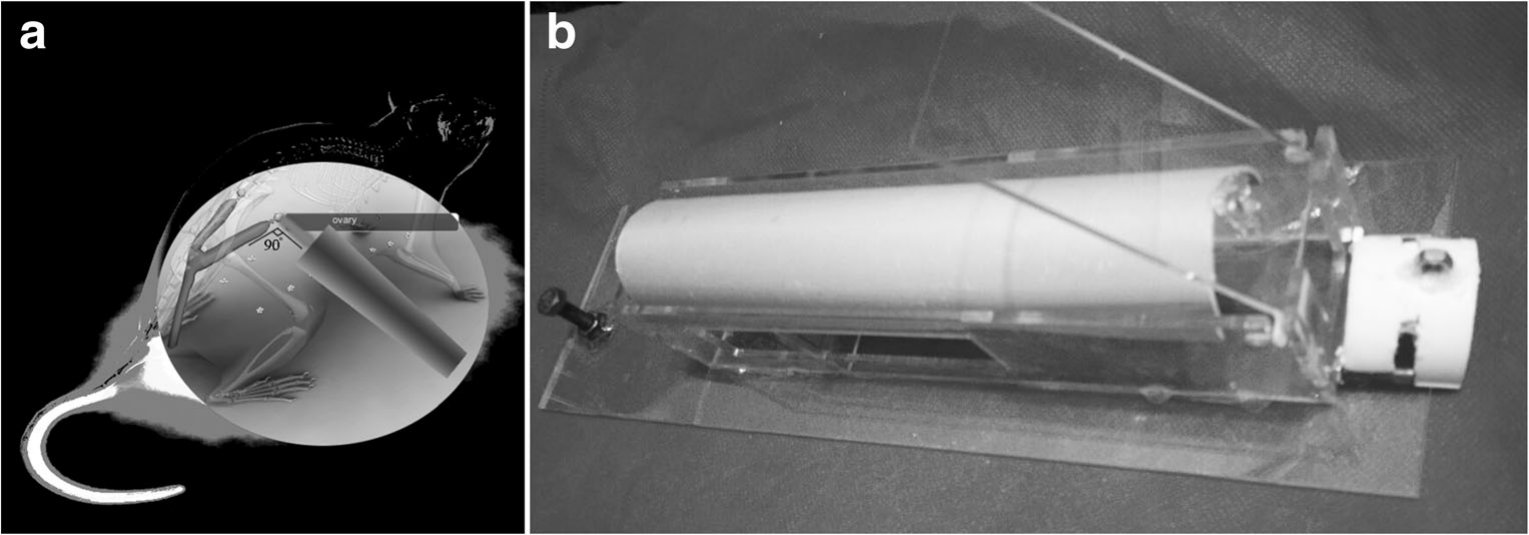
**a** Laser irradiation: This image shows style of laser irradiation during the experiment. **b** Rat restrainer: this restrainer designed for this study. It is approved by Ethics and Research Committee of TUMS (code number: TBZMED.REC.1394.238)

Fourteen rats of RL and NIRL groups received laser on the first 2 days of estrous cycle on specified times (each 6 days, for 48 days). Before doing the experiment, dose transfer percent and irradiation time were determined by a primary experiment. The probes were done by two diode lasers (MUSTANG 2000 +, Moscow, Russia), 630 and 810 nm, by 33 ± 2 and 212 ± 2 power output with 10 mm diameter of nuzzle for both probes, the laser beam area was 0.79 cm^2^, and power densities were 41.77 mw/cm^2^ for red laser and 268.35 mw/cm^2^ for NIR laser beams. Continuous pulse laser mode has been found to be optimal for increasing the activity of protein, ATP synthesis, and cell growth [21, 23]. Irradiation was done for 142.2 and 28 s for RL and NIRL groups, respectively. The laser probes were placed directly on the marked part of the skin with perpendicular angle (Fig. 1a). Henceforth, the same dose of laser was transferred on ovary for two groups; however, the percentage of transferred dose and irradiation time was already determined by a primary experiment. Water was used to provide coupling media for matching impedance between the laser probe and skin of rats. During the treatment, they were fixed in a Plexiglas restrainer designed for this study (Fig. 1b). The average dose reaching the target was 5 J/cm^2^ that was found to be optimal for increasing the activity of protein, ATP synthesis, and cell growth [21]. In this study, we introduced the irradiated time for two groups, red and infrared by calculating average dose to be sure how much the average dose has been reached the target. Transcranial low-level laser therapy (LLLT) treatment was done 16 times in 8 weeks. Also, the marked skin area thickness was measured to calculate skin attenuation coefficient by Lambert-Beer equation.

1 $$ I={I}_0{e}^{-\mu x} $$

2 $$ \mu x=\ln \frac{I_0}{I} $$

### Clomiphene treatment

Seven rats in D group received clomiphene drug. This drug can stimulate folliculogenesis cycle and increase sex hormone activation and consequently increase fertility [26]. The applied dose was 1 μg/kg in 1 cm^3^ water solution. They received drug when RL and NIRL groups were undergoing irradiation. It was determined based on previously published protocol about clomiphene application [26].

### Treatment process

Except for the treatments, all conditions were similar for all rats.

After the experiment, rats were weighted again. After an overnight food deprivation, each animal was anesthetized with an intraperitoneal injection of ketamine/xylazine (60/10 mg/kg). Its blood serum was derived, and the ovary was immediately removed and rinsed in a cold saline and weighed. The ovary tissue was divided into two parts: one parts was frozen by liquid nitrogen and stored in −80 °C refrigerator until the biochemical assay and the other part was immersed in 10% formalin, dehydrated in ethanol, cleared in xylene, and embedded in paraffin for histopathological evaluations. After tissue processing steps, several serial sections of ovary (5 μm thicknesses) were prepared and stained by hematoxylin and eosin (H&E) for microscopic observations and studies. The thickness of media tunica was measured using Motic Images version 2.0 and light microscope. The stained sections were anonymously evaluated by a histologist, see also [27].

The samples were evaluated and analyzed with the statistical, SPSS software. The significance of the data was determined and table and chart were drawn, accordingly.

### Blood serum measurement

Blood samples were taken by cardiac puncher and centrifuged (AZMA Co., Iran-Pars) at 3600 rpm at room temperature for 10 min. Then, they were stored at −80 °C until the assay. They were transferred to veterinary clinic (Dr MUSAVI CLINICE, TABRIZ, IRAN, Dr. Eshrat Khah et al.) for sex hormone measurements. The kit (LH ELISA KIT, FSH ELISA KIT, E2 AccuBind ELISA, and Progesterone AccuBind ELISA-Pishtaz Teb-Iran) is used for counting the amount of hormones, LH, FSH, estrogen, and progesterone, which influence folliculogenesis cycle to ovulation [28–33].

### Laser beam transmission measurement

Ovary and skin samples were taken and data were analyzed by a statistical program. Also, attenuation coefficient was calculated by Lambert-Beer law. By using that, laser absorption has been carried out to a percentage reaching potential that ovaries were calculated. Each data was obtained by three repetitions. They were compared with the standard information. Skin thickness was measured by a standard caliper (Pittsburgh 6-in. digital caliper with 0.03 mm precision).

### Statistical analysis

The number of different follicles and blood hormone levels, LH, FSH, estrogen, and progesterone was statistically analyzed by the IBM SPSS statistics 19 software.

Once, Tukey’s HSD test and, another time, analysis of variance (ANOVA Post Hot) were used. All data were reported as mean ± SD. Significance level was set at *P* < 0.05.

## Result

For histological investigations, changes in the primary, multi-laminar, secondary, and graffian follicles were studied and for hormonal investigations, follicle stimulation hormones, luteinizing, estrogen, and progesterone hormones were taken into consideration.

### Histological investigations

#### Primary follicle

**Fig. 2:**
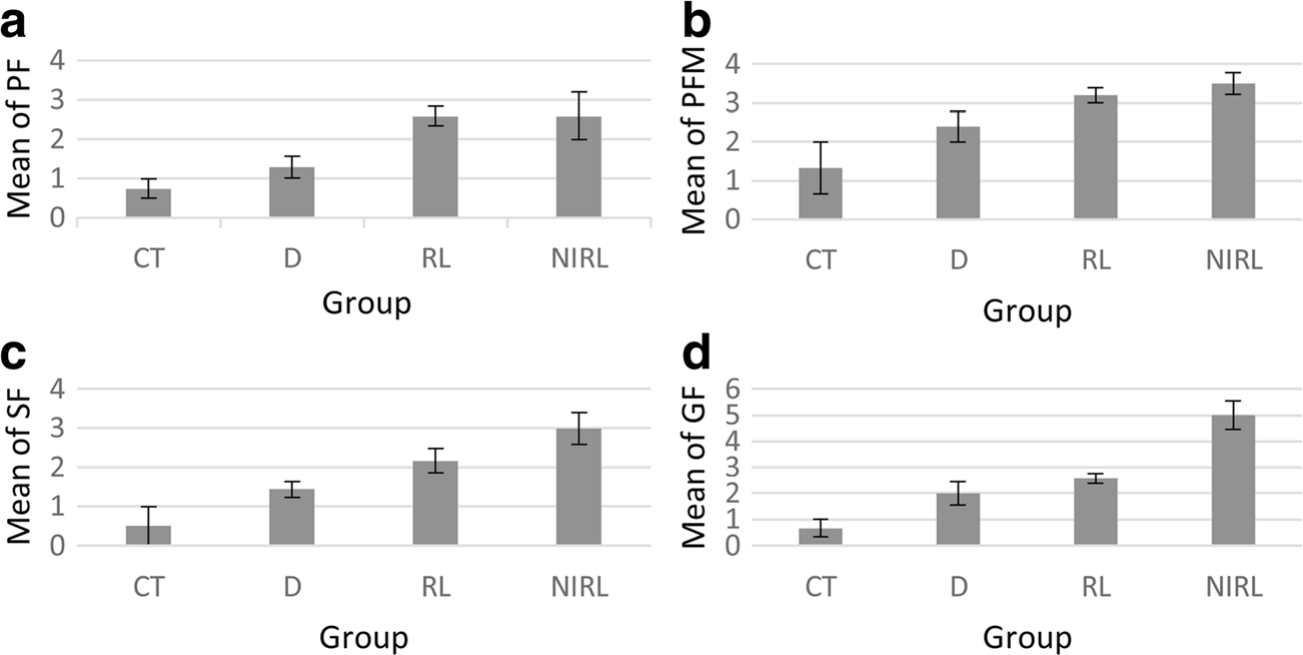
Number of different follicles. **a** PF increase in two RL and NIRL group with equal ratio compared to D and CT group. **b** Increase ratio for PFM is not equal. There is little difference in increase ratio for NIRL and RL group compared with CT and D group apposite of PF number. **c** The changes of SF. It increases in NIRL group more than the other group. **d** The changes of GF. It increases by laser and drug intervention too, but there is not little difference between D and RL group opposites of NIRL. NIRL has the most increase in the number of GF

**Fig. 3:**
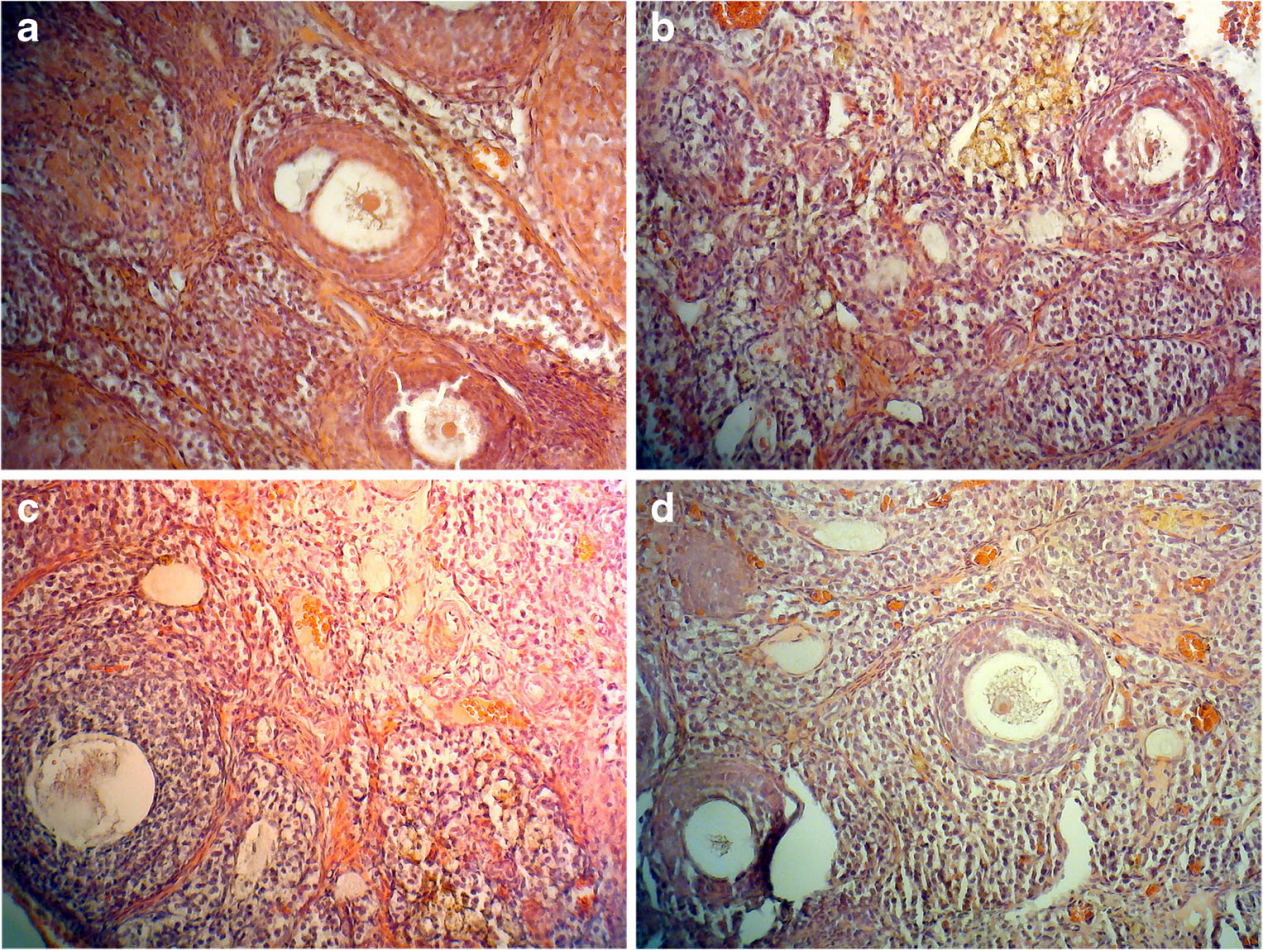
Sections of ovary: there are four parts in this picture. **a** The control picture with less than different follicles. **b** D group. There is an increase in the growth of different follicles compared to CT. **c** RL group which shows that an increase in growth of different follicles more the D and CT group. **d** The changes of NIRL samples which have the most increase in the number of different follicles

The number of PF increased significantly in RL and NIRL groups, compared with CT and D groups. This increase was equal in the RL and NIRL groups (Fig. 2a), and hence, the difference between these two groups was not significant. Also, there was an increase in D group but this growth was less than RL and NIRL groups and did not differ significantly from the CT (Table 1). As can be inferred from the table, increase in the follicle of RL and NIRL groups was approximately 2.02 times more than the D group (Fig. 3).

**Table 1:** Histopathological parameter in ovary tissue

Parameters	CT	D	RL	NIRL
PF	0.75 ± 0.250	1.29 ± 0.286	2.60 ± 0.245*	2.60 ± 0.600*
PFM	1.33 ± 0.667	2.40 ± 0.400	3.20 ± 0.200*	3.50 ± 0.289*
SF	0.50 ± 0.500	1.43 ± 0.202	2.17 ± 0.307*	3.00 ± 0.408*^,^**
GF	0.67 ± 0.333	2.00 ± 0.447	2.57 ± 0.202*	5.00 ± 0.548*^,^**^,^***

Data are presented as mean ± SD

*C* control, *D* clomiphene drug, *RL* red laser injection, *NIRL* near-infrared laser injection

*P < 0.05 as compared with control; **P < 0.05 as compared with drug group; ***P < 0.05 as compared with RL group

So, the increase in the samples of separate groups can be written as

$$ \mathrm{CT}<\mathrm{D}<\mathrm{RL}=\mathrm{NIRL} $$

#### Primary multi-laminar follicles

The results of primary multi-laminar follicles (PFM) were similar to PF analysis (Table 1); the only difference was that the growth ratios in RL an NIRL were not equal. PFM increase in NIRL was approximately 1.46 times more than D and 1.09 times more than RL compared with CT group (Fig. 3). This result has been obtained according to Table 1, and the ascending range of the PFM can be seen in the chart. The highest value was for NIRL group. However, there was not much difference between RL and NIRL while both of them were more than D group, compared with CT (Fig. 2b).

According to the chart, the relationship between the separate groups is as

$$ \mathrm{CT}<\mathrm{D}<\mathrm{RL}<\mathrm{NIRL} $$

#### Secondary follicles

Counting secondary follicles (SF) showed that their number increased significantly in both RL and NIRL groups, compared with CT, while there was not a significant relationship difference between RL and NIRL (Fig. 3). Compared with CT, the increasing rate in NIRL was approximately 1.38 times more than RL and 2.10 times more than D. However, there was no significant difference between D and CT or between D and RL but the difference between D and NIRL was significant (Table 1). The SF production increased almost linearly, with NIRL producing them the most (Fig. 2c).

So, the ordering of groups with respect the number of SF is as

$$ \mathrm{CT}<\mathrm{D}<\mathrm{RL}<\mathrm{NIRL} $$

#### Graffian follicles

The increase in graffian follicles (GF) differed significantly between the CT and RL and between CT and NIRL. Contrary to PF, PFM, and SF results, the difference between RL and NIRL was significant. Also, there was no significant difference between CT and D group, as in other types of follicles. Compared with CT, the increased proportion of GF in NIRL was approximately 2.50 times more than D group and 1.95 times more than RL (Fig. 3). The GF production had an ascending range, like SF and PFM. NIRL samples had maximum amounts and CT samples had minimum amounts. NIRL and RL had increased compared to D and CT. Although there was not much difference between D and RL samples (Fig. 2d), the relationship can be written as

$$ \mathrm{CT}<\mathrm{D}<\mathrm{RL}<\mathrm{NIRL} $$

### Hormonal investigations

#### Follicle stimulation hormone

**Fig. 4:**
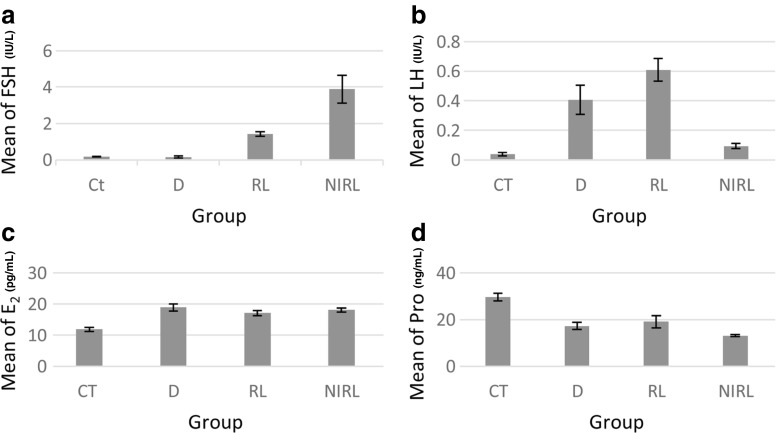
Level of different hormone. **a** An increase in level of FSH in D, RL, and NIRL groups compared to CT. The maximum increase is for NIRL group. **b** Level of LH increase by laser and drug intervention in which maximum increase is for RL group although lowest increase is in NIRL group. **c** An increase in level of E2 in D, RL, and NIRL groups compared to CT while there is not little difference between them. **d** Changes level of Pro in D, RL, and NIRL groups compared to CT. It decreases by intervention and the most decrease is in NIRL group

The level of FSH production has increased in the three experimental groups compared with CT (Fig. 4a). According to the chart, growth in D samples was not much different from CT and there was a large difference between D and IRL. Compared to CT group, NIRL increased approximately 2.73 times more than RL and 25.29 times more than D. On the other hand, the increase of FSH differed significantly between D and RL and between D and IRL groups. This increase was the most for NIRL. Also, RL and the other groups differed significantly (Table 2). According to the table, the growth rate of FSH can be written as

$$ \mathrm{NIRL}>\mathrm{RL}>\mathrm{D}>\mathrm{CT} $$

**Table 2:** The parameter of hormone assay in ovary tissue

Parameters	CT	D	RL	NIRL
FSH (IU/L)	0.16875 ± 0.025174	0.15329 ± 0.052185	1.42025 ± 0.125126^*,**^	3.87733 ± 0.771480^*,**,***^
LH (IU/L)	0.03725 ± 0.011898	0.40557 ± 0.098978^*^	0.60867 ± 0.077197^*^	0.09457 ± 0.018313^**,***^
E_2_ (pg/mL)^f^	11.900 ± 0.7000	18.917 ± 1.1453^*^	17.120 ± 0.8120^*^	18.086 ± 0.6588^*^
Pro (ng/mL)^g^	29.6300 ± 1.57000	17.3050 ± 1.56769^*^	19.1267 ± 2.64739^*^	13.1760 ± 0.41589^*^

Data are presented as mean ± SD

*C* control, *D* clomiphene drug, *RL* red laser intervention, *NIRL* near-infrared laser intervention, *pg/mL* 3.67 pmol/L, *ng/mL* 3.18 nmol/L

*P < 0.05 as compared with control; **P < 0.05 as compared with drug group; ***P < 0.05 as compared with RL group

#### Luteinizing hormone

Increase in the level of luteinizing hormone (LH) differed significantly between CT and D and between CT and RL. But while LH increased in the NIRL, it was not significantly different from CT. Compared to CT group, RL increased LH approximately 1.50 times more than D and 6.44 times more than NIRL. Significant differences existed between D and NIRL and between RL and NIRL, while there was no significant difference between RL and D groups (Table 2). The maximum LH level was for RL and the minimum was for NIRL (Fig. 4b).

The increasing rate for LH level was as

$$ \mathrm{RL}>\mathrm{D}>\mathrm{NIRL}>\mathrm{CT} $$

#### Estrogen hormone

Measurement of estrogen (E_2_) hormone, illustrated that its increase differed significantly between CT and the other groups. Compared to CT, the maximum increase was in D samples (Fig. 4c) and the minimum was in RL and the growth rate in D was proximately 1.11 and 1.05 times more than RL and NIRL, respectively. Meanwhile, there was not much difference in the growth rate of E_2_ between D, RL, and NIRL groups compared with CT group (Table 2). Also, according to the result table, differences between D, RL, and NIRL groups were not significant. So, the trend for E_2_ increase was as

$$ \mathrm{D}>\mathrm{NIRL}>\mathrm{RL}>\mathrm{CT} $$

#### Progesterone hormone

There was significant difference between CT and D, between CT and RL and between CT and NIRL. NIRL group had the most decrease in progesterone (Pro) level and RL had the least. There was not much difference between RL and D samples (Fig. 4d). Ratio of decrease for NIRL was approximately 0.76 times more than D and 0.69 times more than RL group compared whit CT. Compared with D group, Pro decreased in NIRL and increased in RL and CT groups while there was no significant difference between D, RL and IRL groups (Table 2). So, the decreasing rate of Pro can be written as

$$ \mathrm{NIRL}>\mathrm{D}>\mathrm{RL}>\mathrm{CT} $$

#### Attenuation coefficient

**Fig. 5:**
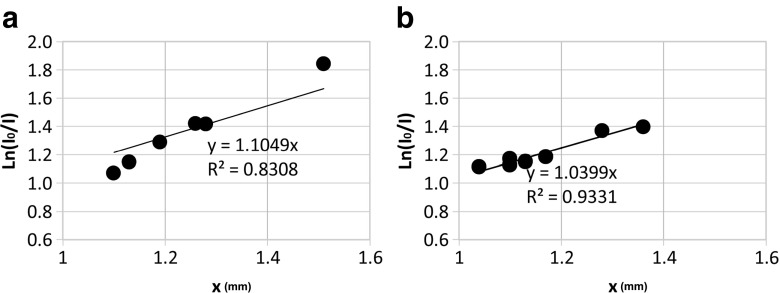
Attenuation coefficient. **a** The attenuation coefficient for red laser by 630 nm wavelength. **b** The attenuation coefficient for near-infrared laser by 810 nm wavelength

The ln (*I*_0_/*I*) (primary and secondary intensity ratio) was plotted to *μx* (*x*: thickness of skin). The attenuation coefficient is obtained from slope of line and presented in the chart; the mean of measurement data is shown in Table 3. At the wavelength of 630 nm, the attenuation coefficient was 1.1049, based on the slope of the line and 1.0879, based on the calculated data for six samples, presented in the table. Additionally, for the wavelength of 810 nm, based on the slope of the line and data calculations for seven samples, it is 1.0299 and 1.0406, respectively (Fig. 5a, b).

**Table 3:** Attenuation coefficient

Wavelength (nm)	*I*_0_ (w/cm^2^)	*I* (w/cm^2^)	ln *I*_0_/*I*	*x* ± 0.03 (mm)	*μ* (1/mm)
630	32.3	7.8	1.4209	1.26	1.12770
	32.7	11.2	1.0715	1.1	0.97410
	31.6	8.7	1.2898	1.19	1.08387
	34.2	8.3	1.416	1.28	1.10625
	33.5	5.3	1.8438	1.51	1.22106
	32.1	10.2	1.1465	1.13	1.01460
				Mean of *μ* = 1.088 ± 0.152	
810	202	50	1.3962	1.36	1.026618
	216	67	1.1706	1.1	1.064182
	188	61	1.1256	1.1	1.023273
	216	55	1.3679	1.28	1.068672
	219	67	1.1844	1.17	1.012308
	195	64	1.1141	1.04	1.07125
	218	69	1.1504	1.13	1.018053
				Mean of *μ* = 1.037 ± 0.138	

Measurement of six samples for red laser by 630 nm wavelength and seven samples for near-infrared laser by 810 nm wavelength

## Conclusion

This study investigated the effect of diode laser at two wavelengths (810 and 630 nm) on folliculogenesis cycle without any surgery and compared the result with result of common drug (clomiphene) application. In the first stage, lasers had positive effect on macroscopic sexual behavior of rats. The effect of both lasers was expected to be positive, but contrary to this expectation, just near-infrared laser was significantly better than drug. It could increase different follicles in folliculogenesis cycle to produce oocyte by increasing the level of useful sex hormones. Rats’ responses to near-infrared laser (NIR group) in producing different follicles at the end of folliculogenesis cycle were approximately 57% positive, 29% neutral, and 14% negative. With red laser (RL group) responses were 43% positive, 14% neutral, and 43% negative. By drug intervention, they were 43 positive, 29% neutral, and 14% negative. Although, the results varied for the two laser modes, the method and statistical analyses that were used in this study showed significant increasing results for NIRL that certainly can be used in future studies for finding a cure to ovarian negligence to produce more oocyte and treat diseases caused by it like PCOS. Hence, this can be investigated on rats with PCOS disease to find certain cure for that.

Compared with previous studies, like someone [9, 17, 20, 21, 23] in which laser was used as a complementary treatment, the present study showed that diode laser with 810 nm wavelength increases the function of folliculogenesis cycle to produce oocyte significantly more than drug.

In interpreting the findings of this study, it is important to take its limitations into consideration. For instance, the exact optical receptor and their optical potential on ovary tissue to produce oocyte are not known. Also, ovary cannot be exactly located due to its small size and its motion on mm range in the body of rat. Additionally, while the result of study was significant and acceptable, the limited sample size bars definitive conclusion. Furthermore, rats were kept in natural environment in the laboratory. However, many potentially important exposure routes were not evaluated. Such exposures include information about dietary intake, blood pressure, and the organs under the skin of irradiated rats, as exposure may vary due to different amounts of tissues such as fat.

The number of ovarian variable follicles in our study exhibited different effects of treatments examined in this research. Given that it is unknown if the ovaries were sick, results are not definite; but based on previous studies [9, 20, 34], our results can be useful in the treatment of hypo activation of ovary.

Finally, this study investigated the direct effect of lasers in near-infrared and red spectrum on producing different follicles in folliculogenesis cycle and did not consider disorders in folliculogenesis cycle to produce oocyte which can later be investigated.

Despite these limitations, this study is an important contribution to previous methods in the treatment of diseases caused by negligence of the ovary. One of these diseases is polycystic ovarian syndrome (PCOS) for which laser had been used as complementary therapy [20, 34]. The aim of this research was to propose the use of lasers autonomously, without any medical and surgical intervention, in the treatment of ovarian negligence in adult women, unlike the past studies [9, 20].
